# Feeling Safe in the Perioperative Period: Experiences from Patients Undergoing Orthopedic Day Surgery

**DOI:** 10.1177/23779608241258562

**Published:** 2024-05-26

**Authors:** Fanny Larsson, Åsa Engström, Silje Rysst Gustafsson, Ulrica Strömbäck

**Affiliations:** Division of Nursing and Medical Technology, 91874 Department of Health Science, Luleå University of Technology, Luleå, Sweden

**Keywords:** feeling safe, day surgery, nursing

## Abstract

**Introduction:**

Day surgery is often preferred by patients, but it can pose challenges in self-management after discharge. In addition, patients undergoing orthopedic surgery report poorer rates of postoperative recovery than patients undergoing general surgery. Understanding patients’ perceptions of feeling safe while undergoing surgery facilitates individualized care and is important since it may affect their recovery.

**Objective:**

The aim of this study was to describe patients’ perceptions of feeling safe in the perioperative period when undergoing orthopedic day surgery under regional anesthesia.

**Methods:**

The design was qualitative and descriptive. Data were collected through a cross-sectional questionnaire containing open-ended questions. Qualitative content analysis with an inductive approach was used for data analysis. Participants’ characteristics were presented descriptively. The study population consisted of a consecutive sample of 97 patients who had undergone orthopedic day surgery under regional anesthesia between March and October 2022.

**Results:**

The categorization process resulted in the development of two categories describing participants’ experience of perioperative feelings of safety when undergoing orthopedic day surgery: having someone near and having a sense of control. The results indicate that the relationship between patients and staff and the perceived feeling of control and participation are factors influencing patients’ perception of feeling safe in the perioperative period.

**Conclusions:**

In perioperative care, nurses play a vital role in fostering patients’ sense of safety by establishing relationships. This ensures that patients can be actively engaged in their own care. Patients also need access to professional and competent staff who strives to add a personal touch and considers their perspective. Since patients undergoing orthopedic day surgery might face a more demanding postoperative recovery than they had initially anticipated, further research is suggested to explore the association between a perceived feeling of safety and postoperative recovery.

## Background

Much of the current safety-related research focuses on patient safety; nonetheless, there is a lack of research on what feeling safe means from the perspective of patients (Mollon, 2014). It is worth noting that while a patient may *be* safe during a procedure, this does not necessarily mean they *feel* safe, and this emotional aspect may hold just as much importance for the individual (Lasiter, 2011; Mollon, 2014). If a person feels unsafe, they may experience feelings like vulnerability, frustration, or anxiety, which may lead to difficulty relaxing, which in turn may complicate the process of recovery (Mollon, 2014; Wassenaar et al., 2014).

### *Review of the Literature*

A common process known as ‘day surgery’ involves admitting a patient, performing a surgery, and discharging them on the same day (Darwin, 2016). In Sweden, almost 2.4 million surgeries were performed in 2021; of these, 1.9 million (80%) were performed as day surgery (Swedish Association of Local Authorities and Regions, 2022). The advantages to day surgery include early mobilization, a lower risk of nosocomial infections, and cost-effectiveness (Darwin, 2016). It is also frequently preferred by patients (Halding et al., 2021). However, day surgery also has limitations. For instance, self-management at home can pose challenges: when questions arise after discharge, patients do not know where to turn, and they may feel that the information provided in the perioperative period did not cover the postoperative period (Halding et al., 2021; Larsson et al., 2022).

Patients who undergo orthopedic surgery report poorer rates of postoperative recovery than those who undergo general surgery, both at the beginning of their recovery and one month after surgery (Forsberg et al., 2018). Regional anesthesia is frequently used for orthopedic surgery and has its advantages compared to general anesthesia, as it reduces postoperative discomfort, exhaustion, and vomiting (Nilsson et al., 2019). However, patients receiving regional anesthesia may have anxiety related to being awake or a fear of witnessing what the surgeon is doing (Stamenkovic et al., 2018). The presence of staff and contact with them—both in the operating room as well as in the post-anesthesia care unit (PACU)—are important to promoting a sense of safety in patients undergoing surgery under regional anesthesia (Bergman et al., 2012; Larsson et al., 2022).

Understanding a patient's perception of feeling safe is important since it may affect their postoperative recovery (Dahlberg et al., 2018). Feeling safe from patients’ perspective has been studied in different contexts (Mollon, 2014; Péculo-Carrasco et al., 2020; Pelto-Piri et al., 2019; Silverglow et al., 2021; Wassenaar et al., 2014), but to the best of our knowledge, research on this topic in a perioperative setting is limited.

The concept of feeling safe in a perioperative context has been defined as not feeling worried or threatened, and it comprises participation, control, and presence. *Participation* includes the patient's desire to take an active part in the decision-making and to impact their own care. *Control* refers to the patient's ability to trust the staff and their competence, as well as the feeling that it is safe to entrust themselves to the care of the staff. *Presence* involves the presence of family members as well as staff, ensuring that the patient does not feel abandoned (Larsson et al., 2023).

Taking patients’ experiences into account is important, and giving primacy to patients’ perspectives may be essential for several reasons. For one, understanding patients’ perspectives when undergoing surgery facilitates individualized care and the identification of areas that require improvement. A positive surgical experience may impact treatment outcomes for patients (Gobbo et al., 2020), and patients perceiving a sense of safety during the perioperative period can influence their postoperative recovery (Dahlberg et al., 2018). Therefore, it is reasonable to assume that a patient's perception of feeling safe plays a crucial role in their overall surgical experience. Given these aspects, the aim of this study was to describe patients’ perceptions of perioperative feelings of safety when undergoing orthopedic day surgery under regional anesthesia.

## Methods

### *Design*

The study design was qualitative and descriptive. Data were collected using a cross-sectional questionnaire containing open-ended questions. The data analysis followed the method of qualitative content analysis as described by Graneheim and Lundman (2004) and Graneheim et al. (2017). Participants’ characteristics were presented descriptively. The reporting of this research follows the guidelines in the consolidated criteria for reporting qualitative research checklist (COREQ) (Tong et al., 2007).

### *Participants and Setting*

This study's population was recruited via consecutive sampling of patients from two different hospitals who had orthopedic day surgery under regional anesthesia between March and October 2022. Inclusion criteria required that the participants had orthopedic day surgery under regional anesthesia and were 18 years old or older. Patients under the age of 18 were excluded because the focus of the study was to describe adult patients’ experiences, and in Sweden, people under the age of 18 are seen as children. The first hospital is a small county hospital in northern Sweden; the ward performs only elective surgeries, mainly orthopedic, both inpatient and day surgeries. Approximately 20–30 day surgeries are performed each week, many of them with regional anesthesia (although general anesthesia is also used). The second hospital, a county hospital in mid-Sweden, performs orthopedic day surgeries each week, as well as both elective and emergency surgeries in different surgical areas. The number of orthopedic day surgery operations that are performed varies each week. The decision to include these two hospitals was based on consideration of the fact that they are in two different geographical areas and that they differ a bit in terms of size and of types of surgeries conducted; with the inclusion of two different hospitals, the variety in experiences may be greater.

For both surgical wards, all patients undergoing orthopedic day surgery typically have an appointment with an orthopedist before the surgery. On the day of the procedure, patients encounter the same staff at the PACU before and after surgery. The PACU staff comprises nurse anesthetists and licensed practical nurses. Anesthesiologists meet the patients at the surgical ward when they induce anesthesia. In the surgical ward, patients also meet nurse anesthetists, operating room nurses, and licensed practical nurses. After their surgery, patients remain in the PACU during phases I and II of their recovery. Before being discharged from the PACU, patients meet their operating orthopedist. For most patients, there is no subsequent follow-up after discharge.

### *Data Collection*

Approval was obtained from the head of the surgical wards to collect the contact information of patients meeting the inclusion criteria from surgical planning documents. In total, 232 patients matched the eligibility criteria and were invited to participate in the study. A questionnaire was sent to the invited patients’ home addresses approximately three weeks postoperative, with an invitation letter about the study's aims and procedures. Patients willing to participate filled out the questionnaire and sent it back to the first author (FL); accordingly, a filled-out questionnaire was seen as informed consent. After two weeks, reminders were sent to the patients who had not returned the questionnaire. Of the 232 patients invited to participate, 97 response envelopes were received, resulting in a response rate of 42%.

The questionnaire contained open-ended questions addressing patients’ experiences regarding their feelings of safety and day surgery (see Table 1). The questionnaire also contained the Feeling Safe During Surgery Scale (FSS) (Larsson et al., 2021) and the Swedish version of the Postdischarge Surgical Recovery scale (S-PSR) (Berg et al., 2010). The quantitative data of the FSS and the S-PSR will be statistically analyzed and reported in a separate study, one with a different aim and with a larger sample size. In addition to the above items, the questionnaire contained nine questions concerning the background variables of age, gender, education level, employment, marital status, type of surgery, previous surgical experience, hospital, and use of sedatives during surgery.

**Table 1.** Open-Ended Questions.

Please describe what “feeling safe when undergoing surgery” means to you?
If you felt safe during your surgery:
Please, describe what was important for you to feel safe before your surgery.
Please, describe what was important for you to feel safe during your surgery.
Please, describe what was important for you to feel safe after your surgery.
If you did not feel safe during your surgery:
Please, describe what contributed to that feeling.

### *Ethics*

The Ethical Review Board in Sweden (Dnr 2020-04703 and 2022-01246-02) approved the study. Permission to conduct the study was received from the unit managers of the surgical wards before data was collected. Invited patients were granted confidentiality and were provided with written information regarding the study's aim and procedures; this written information stated that participation was voluntary and that participants had the freedom to withdraw at any time, without the need for explanation and with no repercussions. The data was encoded and kept in a locked folder on a computer belonging to the first author (FL).

### *Data Analysis*

The responses from the open-ended questions were analyzed using qualitative content analysis (Graneheim & Lundman, 2004; Graneheim et al., 2017) taking an inductive approach. The goal of the analysis was to gain insight into participants’ experiences regarding their perceived sense of safety during orthopedic day surgery. The unit of analysis was defined as the answers to the open-ended questions; it was read multiple times to get a sense of the whole, and meaning units offering information relevant to the study's aim were identified. Subsequently, these meaning units were condensed and categorized through a process involving several steps of merging similar content to create broader categories. In the qualitative content analysis, a *category* describes the data on a manifest level with little interpretation (Graneheim et al., 2017). Throughout the analysis, various categories were tested until a consensus was reached among all authors to ensure alignment with the data; consequently, the data analysis generated two categories. All authors actively participated in the data analysis and the formulation of the final categories. An example of the process of analysis is provided in Table 2.

**Table 2.** Example of the Process of Analysis to Describe the Experience of Feeling Safe Perioperatively.

Meaning unit	Condensed meaning unit	Code	Categorization	Final category
*I had a nurse anesthetist next to me during the entire surgery. She informed me about what was happening continuously.* (Participant 28)	To have a nurse nearby	Nurse nearby		
			To feel close to the staff	
*The fact that I had time to speak to the staff, before the procedure but also during the entire surgery … that made me feel safe* (Participant 50)	Being able to communicate with the staff before and during the surgery	Staff available for conversations		
				Having someone near
*In order to feel safe, I want to meet competent staff that is focusing on performing a professional job* (Participant 63)	Meet competent staff	Competent staff		
			To feel that the staff is competent and humane	
*The staff was being humane and acting compassionately.* (Participant 18)	Staff being humane and showing compassion	Humane staff		

To describe participants’ characteristics, background variables were analyzed descriptively using the Statistical Program for Social Sciences (SPSS) version 28.

The first author has prior experience in empirical research at the master's level and through ongoing doctoral studies. All coauthors are experienced researchers with PhDs as well as specialist nurses. None of the participants had a preexisting relationship with any of the authors. All participants were informed about the study's aim, and their participation was voluntary, with no impact of (non)participation on the care provided by healthcare professionals.

## Results

The age of the participants (*n* = 97) ranged from 22 to 91 years (mean 64.81 years, SD 12.61 years). Participants’ characteristics are presented in Table 3.

**Table 3.** Participants’ Characteristics.

		*N*	%
Gender (*n* = 97)	Men	33	34
	Women	64	66
Level of Education (*n* = 97)	High school	23	23.7
	Upper secondary school	38	39,2
	University < 3 years	15	15.5
	University >3 years	21	21.6
Marital status (*n* = 96)	Single	15	15.5
	Married/partner	71	73.2
	Widow/widower	10	10.3
Type of surgery (*n* = 95)	Hand	28	28.9
	Arm	19	19.6
	Foot	38	39.2
	Shoulder	5	5.2
	Knee	5	5.2
Use of sedatives during surgery (*n* = 97)	Yes	42	43.3
	No	32	33
	Don’t know	23	23.7

In the open-ended questions, the participants described what contributed to or aggravated their perceived feelings of being safe or unsafe during the perioperative period. Through the qualitative content analysis, two categories were developed that described the participants’ perceptions of feeling safe in the perioperative period: having someone near and having a sense of control.

### *Having Someone Near*

The participants described that a perceived feeling of safety was linked to their relationship with the staff and the care provided, and they highlighted their desire to have somebody near, both physically and emotionally. The physical closeness during surgery was described as having someone nearby and not being left alone, thus being able to speak to someone during surgery and with someone who could monitor their well-being during the perioperative period. Some participants emphasized the significance of having a nurse nearby to help them assess any pain or anxiety that might arise and to offer the possibility to be sedated, have analgesics, or, if needed, to convert the form of anesthesia to general anesthesia. Likewise, if participants felt forgotten, this could create feelings of insecurity and being unsafe.

Before my surgery I did not feel safe. I was left alone in a bed for 5 hours, just waiting. A nurse only checked on me twice during that time. I did not even know if I was allowed to go to the toilet. (Participant 56)

I had a nurse anesthetist next to me during the entire surgery. She informed me about what was happening continuously. (Participant 28)

The desire for emotional closeness was expressed as a need to be seen as a person with individual needs. Participants described wanting to know that the staff were not only competent and professional but also human and personal; they wanted the staff to be understanding of their situation and to show empathy and compassion. The participants wished for continuity, so they also wanted to know who they would meet during the perioperative period. When participants trusted the people who cared for them and when the communication was good, they felt safe and were able to hand themselves over into the staff's care.

The staff appeared to be competent, which made me feel safe*.* (Participant 78)

The staff was humane and acted compassionately*.* (Participant 18)

A relaxed, stress-free, and caring environment was another important aspect mentioned by participants. Several described the importance of the staff having time for the patient, along with appearing calm and not stressed. Some participants expressed the desire to be the focus of the staff's attention when undergoing surgery. If the staff was perceived as stressed or if the patient felt that they were not being taken seriously or being spoken to, feelings of being unsafe arose. Similar feelings could also occur when participants felt forgotten while waiting for their surgery.

… that the staff takes the time to talk to me and to explain what they are about to do. (Participant 18)

I felt that the staff were eager to get home as I was having surgery on a Friday. I had intrathecal anesthesia, and it did not wear off until after 5 hours, which made me feel stressed*.* (Participant 34)

### *Having a Sense of Control*

In order to feel safe, participants described a desire to have time to prepare themselves for the procedure, for instance, by getting the appointment scheduled in time. Furthermore, they wanted clear and correct information: knowing what is about to happen and how things are planned helps patients be in charge of their own situation. Likewise, feelings of being unsafe arose if inaccurate or incomplete information was provided. Similar feelings could also arise when participants felt that they were not in control of their situation. Participants expressed a desire to be able to ask questions and actively engage in decision-making throughout the process. Similarly, when they raised questions or wishes, participants wanted the staff to take them seriously and to take their questions into consideration.

I would have felt safe if I had gotten more information on how the procedure would be. Some of the information I got was not even correct*.* (Participant 17)

I want to be able to participate in the care given*.* (Participant 33)

For the recovery process, participants highlighted the importance of knowing the normal trajectory of recovery and whom to turn to if they had any questions after discharge. Support from friends and family postdischarge also promoted feelings of safety: participants felt safer when they had friends or family who provided them with information about their experiences. Some participants mentioned that their previous experience with a similar procedure promoted their feelings of safety. After discharge, feelings of being unsafe may arise when participants do not have anyone to turn to if they have questions, if they do not have a scheduled appointment, if the trajectory of recovery does not occur as expected, or if unexpected pain occurs.

(I want to know) what complications might occur… how I will feel postoperative, who I can contact if I experience anxiety, pain, or any other things. To have follow-up-appointments scheduled beforehand*.* (Participant 17)

Previously, I had surgery on my left foot, so I had some experience. Therefore, I felt safe this time when I was having surgery on my arm*.* (Participant 9)

## Discussion

The aim of this study was to describe patients’ perceptions of perioperative feelings of safety when undergoing orthopedic day surgery under regional anesthesia. The results indicate that the relationship between patients and staff and the perceived feeling of control and participation are factors influencing patients’ perception of feeling safe in the perioperative period.

A wish by the patients to be seen as individual people with unique needs, together with their relationship with the staff, is highlighted in this study's results. Previous research has reported patients’ desire to be treated as individuals, that caring behavior on the part of the nursing staff is important for patients to feel safe (Abelsson & Nygårdh, 2020; Corless et al., 2023), and that the relationship between nurses and patients has a significant impact on patients’ experiences while undergoing surgery (Thoen et al., 2024). In line with Edvardsson et al. (2017) who concluded that staff competence is one of the most important aspects of patients’ experiences of nursing care, the participants in this study highlighted the importance of trusting that the staff is competent and professional. However, in addition, the participants in the present study also emphasized the importance for the staff to appear as people who can show empathy and compassion. Silverglow et al. (2021) studied what contributes to a sense of safety for frail older people in their homes; despite the different context, their results also indicate the importance of the staff's competence and their ability to create positive relationships with their care receivers, which can be achieved by (among other things) acting personally and being present in the moment. Udo et al. (2013) emphasized the importance of nurses integrating their personal selves with their professional roles in surgical care, which involves drawing from personal experiences when interacting with patients who are facing existential challenges. By doing this, nurses can better understand patients and offer compassion, even while risking experiencing a personal emotional impact from the patient's story.

A participant's desire to be seen as an individual as well as to encounter staff who are both competent and personal may be seen as being related to Buber's (1923/1994) description of I–Thou relationships. Instead of an objectifying I–It relationship, a true I–Thou relationship occurs when one person sees the other as an equally valuable subject rather than as an object (Buber, 1923/1994). The patients want to be seen as a Thou and not just an It. In addition, they want staff to appear as a Thou to the patients, not just an object (e.g., It); participants wish for an interpersonal relationship with the staff, where they all contribute with their own personality (Buber, 1954/2004). In contrast to patients striving for interpersonal relationships, nurses working in various hospital wards (including surgical wards) have been found to be task-oriented rather than focused on personalized care (van Belle et al., 2020). This discrepancy might not necessarily stem from nurses’ limitations in providing personalized care but rather may reflect an issue at an organizational level. There seems to be a lack of recognition and appreciation for nurses providing patient-centered care, which is crucial for qualitative care (McCabe, 2004).

Furthermore, it can be understood that patients depend on the staff for their care, which implies an imbalance of power. Participants stressed the importance of trusting the staff and feeling safe as prerequisites for entrusting themselves to the hands of the staff. Lögstrup (1956/1992) described how one party holds the other's life in their hands to varying degrees, and the ethical demand is to safeguard the other's life in a manner that best serves them. Thus, it can be presumed that the patient needs to feel safe and to trust the staff in order to be assured that the staff are fulfilling their ethical demand.

Participation—for instance, being able to pose questions and sensing that one's inquiries and concerns are taken seriously—was highlighted in the study's results. In explorations of the concept of feeling safe in a perioperative context, participation and involvement in decision-making were the most frequently mentioned characteristics (Larsson et al., 2023). Participation through shared decision-making could increase the quality of postoperative recovery as well as patients’ satisfaction with their recovery (Jaensson et al., 2019). In a study of the Dutch clinical practice guidelines for oncology treatment, the results indicated that patient preferences were not included in any guidelines; instead, the guideline writers’ own preferences and their assumptions about patients’ preferences determined the recommendations (Gärtner et al., 2019).

Cooper et al. (2023) found a discrepancy in a perioperative setting between how staff perceive their communication with patients compared to how patients perceive it. Staff often believe that they are engaging in a two-way conversation, while patients instead feel that the information is being communicated in a one-way manner. This discrepancy may be aligned with the findings of this study, which revealed that participation is a prerequisite for patients to feel safe. This underscores the importance of assessing patients’ wishes and their perceptions of the care, and not simply taking the staff's assumptions of patients’ preferences. Halding et al. (2021) reached a similar conclusion, saying that patient education after orthopedic day surgery should be tailored to patient-perceived problems and designed in collaboration with the patient; this could, by extension, increase patients’ sense of control and self-efficacy. Similarly, Thoen et al. (2024) suggested that nurses working in surgical settings should prioritize addressing patients’ needs and preferences.

This study also describes how being in control of one's situation was another aspect for the participants feeling safe. A feeling of being in control of the situation could be achieved by clear and accurate information provided throughout the process, as well as getting the plan for one's surgery in a timely manner and knowing where to go if problems arise after discharge. This aligns with findings by Halding et al. (2021), which demonstrated that consulting with a nurse may enhance patients’ self-efficacy and their sense of control when undergoing orthopedic day surgery. Patients undergoing surgery may experience a loss of power because they have limited control over their bodies during anesthesia, leading to a potential loss of autonomy (Cooper et al., 2023). This study's findings do not address the specifics of the information participants received in the perioperative period; however, in a meta-ethnography, the perioperative information provided during day surgery was described as insufficiently individualized, potentially complicating self-care after discharge (Thoen et al., 2024). Similarly, perioperative information has been described as not comprising enough information about the time after discharge, potentially impeding the process of recovery (Larsson et al., 2022). Thus, it may be assumed that accurate perioperative information empowers patients to feel in control of their situation and consequently feel safer.

### *Strengths and Limitations*

In qualitative content analysis, ensuring trustworthiness throughout the process is crucial. To start, it is essential to include a sufficient number of participants to capture variations in and the diversity of the data (Graneheim et al., 2017). Since the written answers in this study were sometimes short and concise, a large sample size was needed to achieve sufficient information, richness, and depth: a sample should be large enough to encompass a variation in experiences but small enough to permit a thorough analysis of the data (Sandelowski, 1995). There are no guidelines as to the point when sample saturation is attained (Holloway & Galvin, 2017); thus, to assess saturation, all authors deliberated on the issue of sampling to redundancy during the data analysis. It was agreed that saturation was attained with the data collected from the 97 participants; data saturation may have been achieved earlier, but all collected data were included to avoid waste of data and to ensure saturation. In addition, to ensure rigor, all coauthors actively participated in the data analysis process and strove for consensus. There are missing data from participants regarding their marital status (*n* = 1) and type of surgery (*n* = 2) which is displayed in Table 3. Nevertheless, the data regarding participants’ (*n* = 97) experiences of feeling safe is complete, therefore data from all participants are included in the analysis despite missing data on participant characteristics.

The selection of meaning units pertaining to the concept under study is crucial to enhancing credibility (Graneheim et al., 2017). To this end, quotes from the open-ended questionnaire in this study have been included in the Results section. The study participants are diverse in age, education level, marital status, and types of surgeries, which enables more aspects of the problem to be highlighted and may increase the study's credibility. The credibility could also be increased by the inclusion of patients from two different hospitals of different sizes in two different regions. Nevertheless, even though the participants vary in age, education level, and marital status, there is a risk of selection bias. According to Florczack (2022), selection bias is almost inevitable in qualitative research, because data collection may be time-consuming and requires the participants to be willing to share personal experiences. Hence, there is a risk that people who felt either particularly safe or particularly unsafe chose to participate in the study.

The participation of slightly more women than men in the study leads to a risk of gender bias. This is a known problem in scientific studies in general as well as in patient-focused nursing research (Galea & Tracy, 2007; Polit & Beck, 2013). In the gender distribution of the study population (*n* = 232), 44% men (*n* = 102) and 56% women (*n* = 130) were invited; this does not fully explain the overrepresentation of women in this study, but it could be a part of it. Another explanation might be that all the researchers in this study are female, and previous research has shown that male participation is higher when the research group contains men (Polit & Beck, 2013).

The response rate for this study was 42%, which might lead to a lower rate of generalizability, although this is a well-known problem in research. DeKoning et al. (2021) concluded that reduced response rates may be a result of so-called “survey fatigue” after a rise of survey distribution during the COVID-19 pandemic. Due to the qualitative nature of this study, we do not seek to generalize its results. Graneheim and Lundman (2004) use the term “transferability” instead of generalizability in the context of qualitative research. Transferability refers to the extent to which the findings of a study can be applied or transferred to another setting. Nurses in both perioperative and other settings may find insights in the provided findings. Descriptions of participants and study setting have been provided to assist readers in determining whether the results are transferable to another setting,

## Implications for Practice

This study increases awareness of patients’ desires to have a relationship with the surgical staff, to feel safe, to participate, and to feel involved in the care while undergoing orthopedic day surgery. With this knowledge, it is possible to design perioperative care that is more in line with patients’ preferences than with assumptions about what patients want. At an organizational level, nurses and other staff should be given the opportunity to provide a level of care that is designed together with patients, to ensure both patient safety and the sense of safety throughout the perioperative period. This can be achieved by, for instance, incorporating patients’ perceptions into existing guidelines, or explicitly highlighting the importance of considering patients’ perspectives.

## Conclusions

In conclusion, one's perioperative feeling of safety is promoted by having someone nearby and by feeling a sense of being in control, which in turn may influence patients’ postoperative recovery. In perioperative care, nurses can play a vital role in fostering patients’ sense of safety by establishing relationships, which ensures that patients can be actively engaged in their own care. Patients also need access to professional and competent staff who strives to add a personal touch and consider the patient's perspective. As patients undergoing orthopedic day surgery might face a more demanding postoperative recovery than they had anticipated, further research is suggested to explore the association between the perceived feeling of safety and postoperative recovery.
